# The Impact of a High-Intensity Functional Training Program on Athletic Performance of Male Amateur Soccer Players: A Randomized Controlled Study

**DOI:** 10.5114/jhk/202312

**Published:** 2025-11-20

**Authors:** Amir Hossein Haghighi, Farshid Ammarlou, Hamid Marefati, João Gustavo Claudino, Hadi Shahrabadi, Filipe Manuel Clemente, Victor Coswig, Daniel Souza, Paulo Gentil

**Affiliations:** 1 Department of Exercise Physiology, Faculty of Sport Sciences, Hakim Sabzevari University, Sabzevar, Iran.; 2 Sports Science, School of Applied Sciences (FCA), University of Campinas (UNICAMP), Limeira, São Paulo, Brazil.; 3 Department of Biomechanics and Sport Engineering, Gdansk University of Physical Education and Sport, Gdansk, Poland.; 4 Institute of Physical Education and Sports, Federal University of Ceara, Fortaleza, Brazil.; 5 College of Physical Education and Dance, Federal University of Goias, Goiania, Brazil.

**Keywords:** physical fitness, team sports, interval training, resistance training, muscle strength

## Abstract

High-Intensity Functional Training (HIFT) is a popular training modality that aims to improve multiple physical fitness and performance components. This study aimed to examine the effects of HIFT on athletic performance of male amateur soccer players. This randomized controlled study was conducted over a six-week period. Athletes were randomly enrolled into HIFT (n = 12) and control (CON, n = 12) groups. The HIFT group replaced part of the specific training with three weekly sessions of HIFT, whereas the CON group participated only in regular soccer training. Before and after the intervention, athletes were assessed for the 20-m sprint (s), bench press strength (kg), squat strength (kg), average anaerobic power (w), the fatigue index (w/s), the change-of-direction sprint (s), VO_2max_ (ml/kg/min), soccer specific dribbling track test travel distance (m), and soccer dribble sprint skill (s). The HIFT group showed greater (p ≤ 0.001–0.018, pη^2^ = 0.229–0.584) improvements when compared to the CON group, for the 20-m sprint, bench press, and squat strength, fatigue index, VO_2max_, and soccer specific dribbling track test traveled distance. The average anaerobic power approached significance (p = 0.051, pη^2^ = 0.162), but was not significantly different between the groups. HIFT may replace part of regular soccer training to improve athletic performance in amateur soccer players.

## Introduction

Soccer is the most popular team sport worldwide and is characterized by high physical demands (Stølen et al., 2005). During a match, players cover 9–14 km (Bradley et al., 2010) and may run approximately 10 km at an intensity close to the anaerobic threshold (Stølen et al., 2005), including ~600–1200 m of high intensity running and ~150–350 m of sprints (Bush et al., 2015). To be successful, soccer players require technical, tactical, and physical skills including muscular strength, aerobic fitness, anaerobic power, and agility (Stølen et al., 2005).

Considering its complex physical demands, soccer training should be designed to improve different performance domains, leading to the development of different training methods (Thapa et al., 2020). High-intensity functional training (HIFT) is a popular training program model, usually performed at high intensity (Wang et al., 2023), which has been shown to improve many components of physical fitness, including cardiovascular and muscular endurance (Jastrzębski et al., 2025; Mcweeny et al., 2020), muscle strength (Mcweeny et al., 2020; Wang et al., 2023), and anaerobic power (Hovsepian et al., 2021; Ma et al., 2025).

Despite its lack of specificity, HIFT may be interesting for soccer players because it involves high-intensity exercises that simultaneously develop many different physical abilities at the same time (Wang et al., 2023). The rationale for this is based on the relationship between general physical fitness test levels and performance on specific soccer-related markers. For example, Pekas et al. (2016) evidenced a positive relationship between anaerobic power and match performance, including high intensity running (r = 0.920, r^2^ = 0.846), sprinting (r = 0.820, r^2^ = 0.672) and total distance (r = 0.530, r^2^ = 0.281) in soccer players. Kokstejn et al. (2019) reported significant associations between fundamental motor skills and dribbling speed (r = 0.60, r^2^ = 0.360), between physical fitness and dribbling speed (r = 0.420, r^2^ = 0.176), and between fundamental motor skills and physical fitness (r = 0.50, r^2^ = 0.250). Boraczyński et al. (2020) showed that elite soccer players with greater lower body strength had better sprint performance (r = 0.510–0.570, r^2^ = 0.260–0.325), and reported large correlations between absolute peak power and the 1RM half-squat (r = 0.674, r^2^ = 0.454), absolute peak power and knee-extensor maximal voluntary isometric contraction (r = 0.827, r^2^ = 0.684), and the distance covered in the 20-m multistage shuttle run and VO_2max_ (r = 0.950, r^2^ = 0.903).

Since soccer performance depends on the development of different physiological demands, and HIFT is aimed at developing multiple physical abilities, this study aimed to investigate the effect of HIFT on athletic performance of amateur soccer players. Considering that time commitment might be an important barrier for physical preparation (Finlay et al., 2021), this study substituted part of the specific soccer training with HIFT so that both interventions would have similar total training duration. Considering that soccer performance is associated with different physical abilities that HIFT has been shown to improve, our hypothesis was that HIFT would bring additional benefits when compared to specific training alone.

## Methods

**Figure 1:**
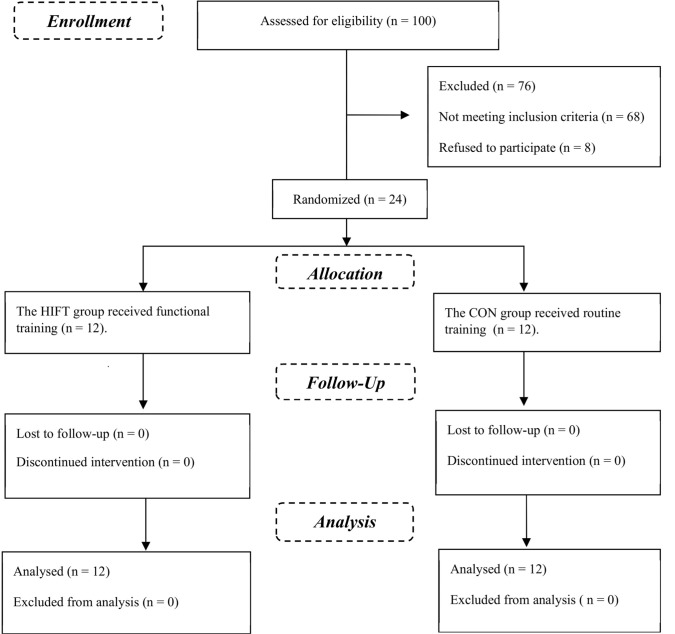
Study flow. HIFT: High intensity functional training; CON: Control

This study adhered to the Consolidated Standards of Reporting Trials (CONSORT) guidelines for reporting randomized controlled studies (Bennett, 2005). The study flow is shown in Figure 1.

### Study Design

The current study followed a randomized controlled study design. The sampling strategy was made by convenience. The eligibility criteria were: i) male soccer players aged 18–31 years, to include players at the age of peak performance (Kalén et al., 2019); ii) amateur athletes with ≥ 3 years of experience in provincial soccer leagues; and iii) no injuries reported in the six months before the start of the study. The exclusion criteria were: i) injury during training, ii) absence in the baseline and/or post-test sessions, iii) not completing the training protocol, and iv) missing ≥ 20% of training sessions. The randomization process was performed based on simple randomization using opaque envelopes after the baseline assessments. The assessors and supervisors were not blinded to group allocation.

### Sample Size Estimation

The sample size (n = 24) was estimated using G*Power software v3.1.9.4, with an alpha coefficient of 0.05, statistical power of 0.80, and an effect size of 0.52, based on linear sprint data from previous research (Bianchi et al., 2023).

### Participants

One hundred amateur adult male soccer players (i.e., Tier 2: trained/developmental based on McKay et al. (2022)) were registered for the study from the Super League of Khorasan Razavi Province, Iran.

This study was approved by the ethics committee of the Hakim Sabzevari University, Sabzevar, Iran (approval code: IR.HSU.REC.1401.003; approval date: 18 April 2022). The study protocol was in accordance with the latest version of the Declaration of Helsinki. Before the start of the intervention, all participants were informed about the purpose of the study, experimental procedures, and possible risks and benefits. Written informed consent was obtained from all participants.

### Methodological Procedures

All procedures were conducted during the pre-season period. After collecting baseline information, participants were divided into two groups: HIFT and control (CON), using a simple random method (lottery). Each of the 24 participants was assigned a number between 1 and 24, and these numbers were randomly selected and placed alternately in two boxes related to the HIFT and CON groups. The randomization process was performed by one of the assessors and concealed until the interventions were assigned. Table 1 shows that there were no significant differences in baseline age, height, body mass, BMI, and training history between the groups (*p* > 0.05). The intervention lasted six weeks, and all tests were performed before the start of the intervention and 48 h after the last training session. All tests were performed by two experienced assessors according to a specific schedule.

**Table 1:** Participants' descriptive data at baseline, presented as mean ± standard deviation.

Variables	HIFT (n = 12)	CON (n = 12)	*p*-value
Age (yr)	24.33 ± 2.70	25.00 ± 3.13	0.583
Body height (cm)	178.16 ± 7.76	179.41 ± 4.90	0.642
Body mass (kg)	72.64 ± 7.17	75.77 ± 5.76	0.251
BMI (kg/m^2^)	22.89 ± 2.56	23.50 ± 1.83	0.504
Training experience (yr)	12.33 ± 2.67	13.00 ± 3.19	0.585

HIFT: High intensity functional training; CON: Control

On the first day, participants were evaluated by a specialist physician and completed a demographic questionnaire. The research was presented, and informed consent was obtained. The second day involved body height and mass measurements, and testing for bench press strength, squat strength, and anaerobic power. Tests for the 20-m sprint, change-of-direction speed, and VO_2max_ were conducted on the third day. Participants rested on the fourth day, and then soccer dribble sprint skill and soccer-specific dribbling track test traveled distance were measured on the fifth day. Two days after the last training session, all the procedures were repeated in the same order, excluding the first day.

A specialist physician assessed each participant for enrolment in the testing and intervention procedures. All tools used to collect data were appropriate for the participants’ conditions, and all safety considerations were respected. Participants were familiarized with the tests in the preceding week. Before the tests, players warmed up (e.g., walking, jogging, stretching) for 10 min at low intensity before starting the physical fitness and soccer-specific tests. A stopwatch (model DS-013, QandQ, China) and a heart rate chest monitor (model PM100, Germany) were used to control the time and intensity of the exercise and tests, respectively.

### Anthropometric Measurements

Body height and mass were measured with a SECA device (Model Seca 714, Seca Vogel and Halk Gmbh) with accuracy of 1 mm and 0.1 kg, respectively. Participants wore light clothing and were barefoot during measurements. The body mass index (BMI) was calculated as body mass(kg)/height(cm)^2^.

### 20-m Sprint

After two practice trials, participants performed two maximal 20-m sprints from a standing position, with a 5-min rest interval between attempts. The best results were used in the analysis (Fernández-Galván et al., 2021). The test ICC was 0.94 (95% CI 0.01, 0.99).

### Muscular Strength

The bench press and squat tests were used to assess upper and lower body muscular strength, respectively, with the ICC of 0.95 (95% CI 0.41, 0.99) and 0.96 (95% CI 0.87, 0.99), respectively. Loads were initially adjusted based on participants’ reports and training history to allow performance of no more than 10 repetitions. When the number of repetitions was greater than 10, the load was increased and another attempt was performed after 5 min. The Brzycki equation was used to estimate their one-repetition maximum (1-RM) (Nascimento et al., 2007): Brzycki Equation = weight lifted (kg)/ [1.0278 – (0.0278 × the number of repetitions performed)].

### Running Anaerobic Sprint Test (RAST)

The running anaerobic sprint test (RAST) was performed on a soccer pitch. The test consisted of 6 × 35-m maximal sprints interspersed with 10-s passive recovery periods. Each sprint began from a standing start position. Anaerobic power, average anaerobic power, and the fatigue index were calculated using the following equations (Redkva et al., 2018):

- Anaerobic power = (body mass × distance^2^)/time^3^
- Average anaerobic power = the sum of the anaerobic power of 6 stages/6
- Fatigue index = (maximum power – minimum power/the sum of the time of 6 stages)

The ICCs was 0.92 (95% CI 0.74, 0.98) for average anaerobic power and 0.91 (95% CI 0.64, 0.97) for the fatigue index.

### Change-of-Direction Sprint

The change-of-direction sprint was measured using a T-test (Pauole et al., 2000) with the ICC of 0.91 (95% CI –0.02, 0.98). On command, participants moved 9.14 m forward to the center cone as quickly as possible, shuffled 4.57 m to the left cone, then shuffled 9.14 m to the far-right cone, and then shuffled 4.57 m to the left of the center cone. Participants then moved backwards as quickly as possible to cross the finish line. The time taken to complete each trial was recorded in seconds.

### VO_2max_ Test

A 20-m track was used to estimate VO_2max_ (St Clair Gibson et al., 1998). Participants were instructed to run between 20 m, as marked by cones, while keeping the pace with the audio signals emitted by the speakers. The initial speed was 8.5 km/h and was increased by 0.5 km/h per minute. The test was terminated when participants failed to reach the end lines concurrent with the audio signals on two consecutive occasions or when participants stopped because of fatigue. The maximal speed that the participant completed in the previous stage was used to estimate VO_2max_ using the following equation: VO_2max_ = (6 × maximal speed) − 24.4. The test ICC was 0.89 (95% CI 0.64, 0.97).

### Soccer Specific Dribbling Track (Hoff Test)

The test was performed in accordance with the instructions provided by Hoff et al. (2002). Briefly, participants dribbled the ball through the cones and lifted the ball over 30-cm high hurdles. Between points A and B, participants moved backward while controlling the ball. Participants performed two four-minute intervals, separated by a three-minute rest interval. The total distance covered in these two efforts was considered for further analyses. The test ICC was 0.83 (95% CI 0.52, 0.95).

### Soccer Dribble Sprint Skill

The slalom dribbling test was used to measure the soccer dribble sprint skills (Gouvea et al., 2016). Participants dribbled by zigzagging between six cones positioned every 2 m along a straight line of 11 m. The test was performed twice and the best results were used in the analysis. The test ICC was 0.91 (95% CI 0.49, 0.98).

### Training Program

The total training times were 3420 and 3240 min for HIFT and CON groups, respectively. The CON group performed six soccer sessions from Saturdays to Thursdays. The HIFT group performed three 90-min soccer sessions on Sundays, Tuesdays, and Thursdays. Three 90–110- min HIFT sessions were conducted on Saturdays, Mondays, and Wednesdays. The HIFT program is presented in Table 2. The program was designed to develop muscle endurance, power, and strength in the same session, using exercises for the upper body, for the lower body, and running with different volumes and intensities. Each session was divided into three consecutive stages with a 15-min rest interval between them (Schlegel, 2020). The regular soccer training program included technical exercises (e.g., dribbling skills, shooting, and heading), tactical exercises (e.g., attack and defense tactics), and games. The warm-up and cool-down phases lasted 10–15 and 5 min, respectively. The warm-up included running forward and backward, single-leg toe touches, dynamic hamstring stretching, hip rolls, and knee-up exercises. The cool-down consisted of low-intensity exercises such as slower walking, jogging, and stretching.

**Table 2:** High intensity functional training program.

First stage (lower body)									
			Exercise load						
Exercises	Repetitions	Circuits	Week 1	Week 2	Week 3	Week 4	Week 5	Week 6
Clean	5	5 in 5 min	1/3 BW	1/3 BW	1/2 BW	1/2 BW	2/3 BW	2/3 BW
Squat	10							
Deadlift	15							
15 min of rest									
Second stage (upper body and midsection)									
			Duration						
Exercises	Repetitions	Circuits	Week 1	Week 2	Week 3	Week 4	Week 5	Week 6
Pull up	5	As many as possible	10 min	10 min	12 min	12 min	14 min	14 min
Sit up	10							
Push up	5							
Back extension	10							
15 min of rest									
Third stage (high intensity running)									
Exercises	Repetitions	Sets	Week 1	Week 2	Week 3	Week 4	Week 5	Week 6
Running	See weeks column	See weeks column	5×1000 m (70–75% HR_max_)	2×100 m (all-out)	1×200 m (all-out)	2×200 m (all-out)	4×100 m (all-out)	10×10 m (all-out)
				2×400 m (all-out)	1×800 m (all-out)	2×400 m (all-out)	2×200 m (all-out)	10×20 m (all-out)
				4×1000 m (70–75% HR_max_)	3×1000 m (75–80% HR_max_)	1×800 m (all-out)	1×400 m (all-out)	5×60 m (all-out)
						2×1000 m (75–80% HR_max_)	1×800 m (all-out)	6×100 m (all-out)
							1×1000 m (80–85% HR_max_)	4×200 m (all-out)
								1×1000 m (80–85% HR_max_)

BW: Body weight; HR_max_: Maximum heart rate * During the execution of the exercise protocol, the average maximal number of circuits as possible in 10, 12 and 14 min was 5, 6 and 7 circuits, respectively

### Statistical Analysis

The dependent variable values were reported as means and standard deviations. The Shapiro-Wilk test was used to determine the normality of the data. The within-subject reliability of physical fitness and soccer-specific tests was assessed using the intraclass correlation coefficient (ICC). A paired-sample *t*-test was used to compare within-group pre- and post-intervention changes. The effect size (*d*) was calculated using the following method for paired samples: mean baseline scores minus mean follow-up scores divided by the pooled baseline standard deviation. Following Cohen (1988), *d* = 0.20, *d* = 0.50, and *d* = 0.80 represented small, medium, and large effect sizes, respectively. A mixed ANOVA was used to determine the effectiveness of the intervention over time (group × time). The effect size of the independent samples was calculated using partial eta squared (pη^2^). An effect size of 0.10 was considered small, 0.25 medium, and 0.40 large (Cohen, 1988). Data analysis was performed using SPSS software (version 26, SPSS Inc., Chicago, Illinois, USA). Statistical significance was set at *p* < 0.05.

## Results

**Figure 2:**
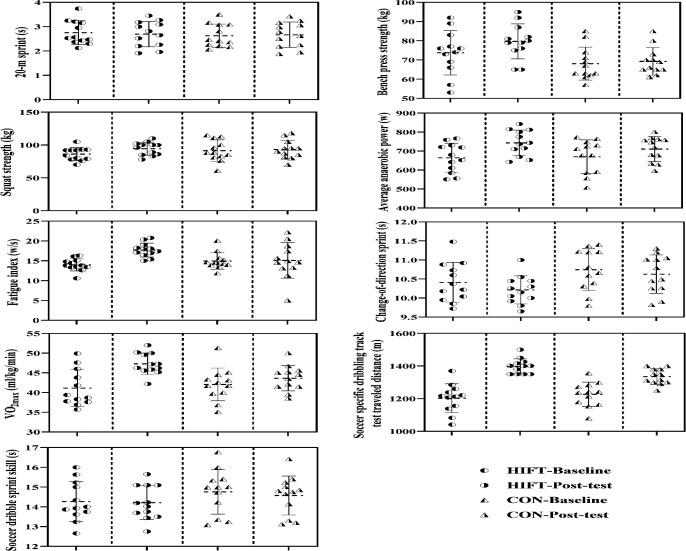
. The mean and standard deviation of the data related to physical fitness and specific soccer indicators of soccer players. HIFT: High intensity functional training; CON: Control

All participants in both the CON and HIFT groups who were present at baseline completed the training protocol without any relevant reports of injury, pain or discomfort. The test results are shown in Figure 2.

### 20-m Sprint

The time to complete the 20-m sprint decreased significantly by −4.73% (*t* = 6.934, *p* < 0.001, *d* = 2.002) for the HIFT and −1.11% (*t* = 16.583, *p* < 0.001, *d* = 4.789) for the CON group. Between-group analysis revealed that the values were significantly different (*p* < 0.001, pη^2^ = 0.584).

### Bench Press Strength

Bench press strength increased significantly by 8.01% (*t* = −7.377, *p* < 0.001, *d* = 2.129) for the HIFT group, but the differences for the CON group were not significant from baseline (*t* = −1.432, *p* = 0.180, *d* = 0.413). There were significant differences between groups (*p* < 0.001, pη^2^ = 0.440).

### Squat Strength

Increases in squat strength were significant for the HIFT group (9.68%; *t* = −10.055, *p* < 0.001, *d* = 2.903), but not for the CON group (*t* = −1.089, *p* = 0.299, *d* = 0.314). The comparison between groups revealed significant differences (*p* = 0.001, pη^2^ = 0.425).

### Average Anaerobic Power

The average anaerobic power during the RAST increased by 11.84% (*t* = −10.317, *p* < 0.001, *d* = 2.978) in the HIFT group and 6.03% (*t* = −2.392, *p* = 0.036, *d* = 0.691) in the CON group, with no statistically significant difference between the groups (*p* = 0.051, pη^2^ = 0.162).

### Fatigue Index

The HIFT group showed a significant reduction of 26.59% in the fatigue index during the RAST (*t* = −8.712, *p* < 0.001, *d* = 2.515), while there were no significant changes in the CON group (*t* = −0.122, *p* = 0.905, *d* = 0.035), with significant differences between groups (*p* = 0.007, pη^2^ = 0.288).

### Change-of-Direction Sprint

The change-of-direction sprint improved for the HIFT (−1.92%; *t* = 3.784, *p* = 0.003, *d* = 1.092) and CON (−1.12%; *t* = 5.470, *p* < 0.001, *d* = 1.579) groups, with no significant differences between the groups (*p* = 0.175, pη^2^ = 0.082).

### VO_2max_

VO_2max_ significantly increased by 15.01% (*t* = −8.645, *p* < 0.001, *d* = 2.495) in the HIFT group and by 3.73% (*t* = −3.686, *p* = 0.004, *d* = 1.064) in the CON group, with significant differences between the groups (*p* < 0.001, pη^2^ = 0.583).

### Soccer Specific Dribbling Track

The HIFT and CON groups significantly improved soccer-specific dribbling by 16.24% (*t* = −6.605, *p* < 0.001, *d* = 1.907) and 8.89% (*t* =−6.582, *p* < 0.001, *d* = 1.899), respectively. Comparisons revealed significant differences between the groups (*p* = 0.018, pη^2^ = 0.229).

### Soccer Dribble Sprint Skill

Only the CON group showed improved soccer dribble sprint skills (−1.22%; *t* = 3.663, *p* = 0.004, *d* = 1.057 vs. −0.42%; *t* = 0.910, *p*= 0.382, *d* = 0.263), although there were no differences between the groups (*p* = 0.143, pη^2^ = 0.095).

## Discussion

The present study showed that replacing three weekly sessions of specific soccer training with HIFT led to improvements in most of the tests performed, with higher results for the sprint, muscular strength, the fatigue index, aerobic power, and soccer-specific dribbling track in comparison to the group that performed regular soccer training only.

Dransmann et al. (2021) did not report improvements in sprint performance after HIFT. However, their training protocol did not involve high-velocity running, which highlights the importance of training specificity. Improvements in sprinting might be of practical importance for soccer, since a match involves 20–41 sprints, depending on the player’s position (Gregson et al., 2010) and high-speed activity accounts for approximately 8% of the total distance covered during a match (Rampinini et al., 2007), with numerous turns, runs with directional changes, accelerations, and decelerations (Matlák et al., 2016). Considering that these high-intensity activities are associated with crucial defensive and offensive actions, increases in physical performance may positively affect the game outcomes.

Increases in muscular strength, as measured by the bench press and squat exercises, seen in the present study are in line with those previously reported by McWeeny et al. (2020) in active adults. The increases in muscular strength as a result of HIFT seem to be consistent (Wang et al., 2023) and related to the performance of high-intensity resistance training (Posnakidis et al., 2022). Specifically to soccer, Villaseca-Vicuña et al. (2021) found a significant relationship between 1RM squats and total distance covered in official matches. Players with greater muscular strength may have a greater capacity to transfer it to high-intensity actions in real competitive situations (Boraczyński et al., 2020). Additionally, greater muscular strength may reduce muscle damage following match play (Owen et al., 2015), thus favoring recovery and performance over consecutive matches.

The increases in anaerobic power and the fatigue index for the HIFT group might be relevant to soccer, since anaerobic power is important during short-duration high-intensity activities such as shooting, passing, and jumping (Nikolaidis, 2014). Moreover, improvements in fatigue resistance may allow these tasks to be performed at higher levels over a match. Previous studies involving HIFT have been controversial in these respects. Adami et al. (2022) reported that individuals following HIFT exhibited increased anaerobic power performance. Moreover, HIFT has been shown to improve anaerobic power and the fatigue index in basketball players (Hovsepian et al., 2021). In contrast, Caloglu and Yüksel (2020) showed that the anaerobic power of wrestlers did not change significantly in response to HIFT. This divergence may be due to the general program design. McWeeny et al. (2020), showed that a lack of improved anaerobic capacity might be associated to excessive accumulated fatigue.

The increase in VO_2max_ in soccer players may improve match performance (e.g., distance covered, the number of sprints, the number of actions with the ball) (Helgerud et al., 2001). Soccer specific dribbling track is a test designed by Hoff et al. (2002) that measures VO_2max_ in soccer players. A previous study has reported that individuals following HIFT have high VO_2max_ levels which are comparable with endurance-trained subjects and higher than in those that perform resistance training only (Adami et al., 2022). In agreement with this, Lu et al. (2021) reported that HIFT promoted similar increases in VO_2max_ when compared to running. Although we are not aware of any studies investigating the effects of HIFT on soccer-specific dribbling tracks, Arazi et al. (2017) showed that six weeks of high-intensity interval training improved the soccer-specific dribbling track test traveled distance.

Dribbling is one of the most important attacking skills in soccer, and sports clubs seek players who excel (Wilson et al., 2019). However, the present study did not observe a significant change in soccer dribble sprint skills in response to HIFT. Although there were no significant differences between the groups, only the CON group improved dribbling skills, which might be associated with a higher volume of specific training performed by this group.

Despite these benefits, the recommendation to add HIFT sessions to soccer training programs requires some consideration. As HIFT is an autoregulated training program with high levels of motivation, it may be difficult to control the training intensity and regulate recovery (da Silveira Castanheira et al., 2023). In addition, it is important to acknowledge that although no injuries were reported in the present study, HIFT has a relatively high risk of injuries, comparable to other sports (Gardiner et al., 2020). Therefore, considering that soccer players are already exposed to injuries due to their activities, it is important to analyze the cost benefit of adding HIFT over the long term and evaluate whether similar benefits could be obtained with other strategies with lower risks. Meanwhile, additional attention may be needed for injury and overtraining risks, especially because of the high volume of lower-limb stimuli.

This study has some limitations that should be considered. First, the participants, personnel, and assessors were not blinded to the interventions for practical reasons. Second, the short training duration might limit extrapolation to long-term results.

## Conclusions

Notwithstanding its limitations, the present study leads to the conclusion that HIFT might be used as part of a training program for soccer players during the pre-season period, as it brings many benefits related to performance compared to regular soccer training. However, it is important to be careful with its implementation during competitive phases, as physical demands might already be too elevated during these periods.
